# Impact of Coronavirus Disease 2019 (COVID-19) Pandemic on Surgical Site Infection in Patients with Inflammatory Bowel Disease—A Monocentric, Retrospective Cohort Study

**DOI:** 10.3390/jcm13030650

**Published:** 2024-01-23

**Authors:** Rahel Maria Strobel, Amelie Baehr, Ralf Hammerich, Daniel Schulze, Kai Siegfried Lehmann, Johannes Christian Lauscher, Katharina Beyer, Susanne Dorothea Otto, Claudia Seifarth

**Affiliations:** 1 Department of General and Visceral Surgery, Campus Benjamin Franklin, Charité—Universitätsmedizin Berlin, Corporate Member of Freie Universität Berlin und Humboldt-Universität zu Berlin, Hindenburgdamm 30, 12203 Berlin, Germanyclaudia.seifarth@charite.de (C.S.); 2 Department of Clinical Quality and Risk Management, Charité—Universitätsmedizin Berlin, Corporate Member of Freie Universität Berlin und Humboldt-Universität zu Berlin, Charitéplatz 1, 10117 Berlin, Germany; 3 Department of Biometry and Clinical Epidemiology, Charité—Universitätsmedizin Berlin, Corporate Member of Freie Universität Berlin und Humboldt-Universität zu Berlin, Charitéplatz 1, 10117 Berlin, Germany

**Keywords:** COVID-19, surgical site infection, inflammatory bowel disease, postoperative complication

## Abstract

(1) **Background:** Surgical site infections (SSIs) are a relevant problem with a 25% incidence rate after elective laparotomy due to inflammatory bowel disease (IBD). The aim of this study was to evaluate whether stricter hygienic measures during the COVID-19 pandemic influenced the rate of SSI. (2) **Methods:** This is a monocentric, retrospective cohort study comparing the rate of SSI in patients with bowel resection due to IBD during COVID-19 (1 March 2020–15 December 2021) to a cohort pre-COVID-19 (1 February 2015–25 May 2018). (3) **Results:** The rate of SSI in IBD patients with bowel resection was 25.8% during the COVID-19 pandemic compared to 31.8% pre-COVID-19 (OR 0.94; 95% CI 0.40–2.20; *p* = 0.881). There were seventeen (17.5%) superficial and four (4.1%) deep incisional and organ/space SSIs, respectively, during the COVID-19 pandemic (*p* = 0.216). There were more postoperative intra-abdominal abscesses during COVID-19 (7.2% vs. 0.9%; *p* = 0.021). The strictness of hygienic measures (mild, medium, strict) had no influence on the rate of SSI (*p* = 0.553). (4) **Conclusions:** Hygienic regulations in hospitals during COVID-19 did not significantly reduce the rate of SSI in patients with bowel resection due to IBD. A ban on surgery, whereby only emergency surgery was allowed, was likely to delay surgery and exacerbate the disease, which probably contributed to more SSIs and postoperative complications.

## 1. Introduction

In March 2020, the World Health Organization (WHO) declared coronavirus disease 2019 (COVID-19) a global pandemic caused by severe acute respiratory syndrome coronavirus 2 (SARS-CoV-2) [1]. This led to a change in hospital routines worldwide to minimize the risk of infection to healthcare workers and patients. Hygienic measures were tightened, bans on visits were imposed, and prohibitions on surgery were enacted [2,3]. General precautions included social distancing, use of personal protective equipment, surface disinfection, and hand hygiene [1]. Surgery carried out during this period was therefore subject to special conditions. Clinical examinations and wound control took place under increased hygienic standards.

Surgical site infections (SSIs) are a common healthcare-associated complication in surgical patients. Studies describe SSI rates up to 10–26% after colorectal surgery [4,5,6], where SSIs are more frequent than after other surgical procedures. Hence, surgeons have to deal with this significant clinical problem constantly. In particular, patients with inflammatory bowel disease (IBD) have an increased risk of SSI [7,8]. They often present risk factors such as immunosuppression, abdominal abscesses, impaired nutritional status, or intestinal obstruction. Other patient-related risk factors include advanced age, obesity, smoking, and medical conditions such as diabetes mellitus, respiratory comorbidity, or anemia [7,8,9,10]. 

Alteration of most patient-related risk factors—if possible at all—requires long-term modification, which is usually not feasible in urgent intervention for IBD or other colorectal surgery. SSIs contribute to morbidity, mortality, length of hospital stays, and overall costs [11]. In Germany, SSIs result in up to one million additional days of hospitalization per year [12]. In our clinic, costs for hospital stays were approximately 5.450 Euro (USD 6430) higher in patients with SSI compared to patients without SSI [13]. In the United States, additional costs for SSI range between USD 10443 and 25546 [14,15]. 

In contrast to most patient-related risk factors, procedure-related risk factors may be modified by adjustments in the daily routine. Therefore, the assessment of these modifiable procedure-related risk factors is essential to decrease the rate of SSIs and to improve patient safety. In the RECIPE trial (Reduction of Postoperative Wound Infections by Antiseptica), which was conducted at the department for general, visceral, and vascular surgery of the Charité—Campus Benjamin Franklin, we showed a reduction in SSI in elective laparotomies by intraoperative subcutaneous wound irrigation with polyhexanide solution compared to wound irrigation with saline [16]. In IBD patients, laparoscopic procedures offer a potential advantage, especially for frequently immunosuppressed patients, to reduce SSIs [17]. Other studies have shown the influence of avoiding razors for hair removal, decolonization, maintaining normothermia, and perioperative glycemic control [18]. The WHO guidelines for the prevention of SSI include the use of antibiotic prophylaxis, skin decontamination, and hygienic principles such as hand hygiene and asepsis during wound care [19,20]. As these basic hygiene recommendations were endorsed by the implemented COVID-19 regulations, we questioned whether there was a decrease in SSI during the pandemic.

The aim of this study was to evaluate whether the COVID-19 pandemic (between the 1st of March 2020 and the 15th of December 2021) and concomitant preventive measures had an influence on the rate of SSI in patients with bowel resection due to IBD.

## 2. Patients and Methods

### 2.1. Trial Oversight

This is a monocentric, retrospective cohort study that was performed at the Department for General and Visceral Surgery at Campus Benjamin Franklin of Charité–Universitätsmedizin Berlin.

The rate of SSI following small or large bowel resection for IBD during the COVID-19 pandemic (between the 1st of March 2020 and the 15th of December 2021) was compared with a pre-COVID-19 cohort (between the 1st of February 2015 and the 25th of May 2018). This cohort comprised patients with IBD who participated in the RECIPE trial. The RECIPE trial was a single-center, randomized controlled prospective study with two parallel treatment groups, comparing intraoperative subcutaneous wound irrigation with 0.04% polyhexanide solution to 0.9% saline in elective open or laparoscopically assisted visceral surgery [16].

### 2.2. Study Population

Patients who were 18 years or older, capable of giving informed consent, and undergoing elective open or minimally invasive resection of the small or large intestine due to IBD were eligible to participate. Emergency surgery was an exclusion criterion. Patients with a diagnosis of IBD who underwent bowel resection because of another underlying disease such as mesenteric ischemia were not included in this trial. The perioperative patients’ data were taken from the hospital documentation system. Surgeons on the ward examined the incision site of all the patients regularly during the postoperative course. There was a thorough examination of SSI during the hospital stay. As this was a retrospective trial, all the patients were contacted by a self-completed questionnaire regarding their 30-day morbidity and any wound infections. This self-completed questionnaire has already been used and published in the RECIPE trial [16]. In case of uncertain status of the wound, the patient was examined in our outpatient department. 

### 2.3. Perioperative Setting

All the patients received intravenous antibiotic prophylaxis with 1.5 g of cefuroxime (M.P.I. Pharmaceutica, Hamburg, Germany) and 500 mg metronidazole (Braun, Bethlehem, PA, USA) 30 min before surgery. In the operating room, the skin was disinfected with Braunoderm (Braun, Melsungen, Germany) containing 50% 2-propanol and 1% povidone-iodine. Dual-ring wound protectors were used for recovering resected bowel specimens. Subcutaneous wound irrigation with 0.04% polyhexanide solution was performed after closure of the fascia and before skin closure.

### 2.4. Hygienic Measures during the COVID-19 Era at Charité—Universitätsmedizin Berlin, Campus Benjamin Franklin

During COVID-19, we categorized preventive hygienic measures into “mild, medium, and strict” according to the imposed bans. Mild hygienic measures were applied between the 15th of March 2020 and the 19th of November 2020. During that time, only one visitor per patient was allowed. In general, a minimum distance of 1.5 m (4.9 feet) between two people was required inside and outside of the hospital. Hospital staff was advised to wash and disinfect hands regularly, to air the rooms, and to use break rooms alone.

The category “medium” was present in the period between the 20th of November 2020 and the 16th of December 2020 and between the 2nd of April 2021 and the 26th of December 2021. Medium hygienic regulations were specified as the obligation for visitors to wear an FFP 2 mask inside the hospital. After the 26th of April 2021, visitors were only allowed to enter the hospital with a negative COVID-19 test that was conducted within the previous 24 h.

Medium hygienic regulations also included a visiting ban. Only patients who were hospitalized for at least one week were allowed to receive visitors. Exceptions were made for children younger than 16 years and multimorbid patients. 

Strict surgical and hygienic bans existed between the 17th of December 2020 and the 1st of April 2021 and included the performance of only emergency operations at Charité. It was at the discretion of the surgeons to apply a hardship provision for individual patients with justification for the surgery. Furthermore, a visiting ban was enacted between the 7th of January 2021 and the 1st of April 2021. After February 2021, the use of an FFP2 mask was obligatory for the whole staff inside the hospital building. Surgeons were advised to wear FFP2 masks intraoperatively after that date. 

### 2.5. Outcomes

The primary endpoint was the rate of surgical site infection (SSI) within 30 days postoperatively, according to the standardized criteria by the U.S. Centers for Disease Control and Prevention (CDC) [21]. SSIs were categorized as superficial, deep, and organ/space infections. Deep SSIs involved the deep soft tissue around the incision, fascial and muscle layers, and organ/space infections affected any part of the body deeper than fascial/muscle layers that were accessed during the operation (see Table 1). 

The follow-up to this retrospective trial was conducted with a questionnaire that was sent by mail to all the eligible patients. This questionnaire was already established in the RECIPE trail in a standardized manner. The patients were asked whether an SSI occurred within 30 days after the procedure and if readmission to the ward or outpatient treatment was necessary due to an SSI. Patients in the RECIPE trial were prospectively followed for 30 days postoperatively and answered the same questionnaire.

The patients’ characteristics and comorbidities, as well as surgical details, were recorded and reviewed. Wound classification was categorized into clean (I), clean-contaminated (II), contaminated (III), and dirty (IV) as defined by the American College of Surgeons National Surgical Quality Improvement Program [22]. Surgical postoperative complications were documented according to the Clavien–Dindo classification [23]. 

### 2.6. Statistical Analysis 

As most variables showed no normal contribution, the primary outcome was analyzed with a cross-tabulation, chi-squared test for the categorical variables, and a non-parametric test for the continuous variables. A covariate adjustment for the primary endpoint—a rate of SSI 30 days postoperatively—was performed with the following control variables: diabetes mellitus, malignant disease, ASA score, anemia, wound class, site of operation, surgical approach, duration of surgery, intraoperative intra-abdominal abscess, serosal tear, postoperative intra-abdominal abscess, and length of hospitalization. Values of the multivariate analysis were expressed as the odds ratio (OR), 95% confidence intervals (CI), and *p*-value. Due to the skewed distribution of the quantitative variables, group differences were analyzed by the Mann–Whitney U test. Additional parameters were depicted according to their scale and distribution with absolute and relative frequencies for the categorical parameters and median, minimum, and maximum for the quantitative parameters. *p*-values ≤ 0.05 were considered statistically significant. Statistical analysis was carried out using IBM SPSS Statistics 26 (IBM, Armonk, New York, NY, USA).

### 2.7. Ethics

The study was approved by the ethics committee of Charité—Universitätsmedizin Berlin (application number EA4/077/22). The trial was conducted in accordance with the ethical principles of the Declaration of Helsinki and the principles of Good Clinical Practice (ICH-GCP E6) [24].

## 3. Results

### 3.1. Surgical Site Infections during the COVID-19 Pandemic and Pre-COVID-19 

The rate of SSI during COVID-19 was 25.8% compared to 31.8% in the RECIPE trial pre-COVID-19 (see Table 2). There was no significant difference between the two cohorts (*p* = 0.345). Deep incisional SSIs and organ/space SSIs occurred in four (4.1%) patients during COVID-19 compared to two (1.9%) patients pre-COVID-19, respectively (*p* = 0.216). A covariate adjustment for the primary endpoint—rate of SSI 30 days postoperatively—between the two study populations showed no significant risk reduction (OR 0.94; 95% CI 0.40–2.20; *p* = 0.881). 

Due to the study design, there is a difference in subcutaneous wound irrigation between the two study populations. Ninety-seven (100%) patients during COVID-19 were irrigated with 0.04 polyhexanide solution compared to 59 (55.1%) pre-COVID-19 (*p* < 0.001). Therefore, univariate analysis of SSI was performed in patients who underwent elective surgery and subcutaneous irrigation with 0.04% polyhexanide solution before the COVID-19 pandemic and during the COVID-19 pandemic. Overall, there were 156 patients, 59 pre-COVID-19 and 97 during the COVID-19 pandemic. The rate of SSI was nearly the same: 15 (25.4%) patients in the pre-COVID-19 group, and 25 (25.8%) patients during COVID-19 suffered from SSI; *p* = 0.961. 

### 3.2. Baseline Characteristics during the COVID-19 Pandemic and Pre-COVID-19 

We compared only patients with an elective surgery setting and complete follow-up of 30 days postoperatively between the periods of the COVID-19 pandemic (n = 97) and pre-COVID-19 (n = 107). Emergency surgery was performed during COVID-19 in 26 patients (21.1%). Those patients were excluded in the comparative analysis of the primary endpoint. 

Table 3 shows the baseline characteristics. There was no difference in sex (*p* = 0.276), age (*p* = 0.790), or BMI (*p* = 0.179). The patients during COVID-19 had, more often, American Society of Anesthesiology scores (ASA scores) of 3–4 (*p* = 0.021) and malignant disease (*p* = 0.025) compared to pre-COVID-19, whereas patients pre-COVID-19 suffered more often from diabetes mellitus (*p* = 0.045) and anemia (*p* = 0.014). The surgical approach (*p* = 0.479), resected organ (colon or small bowel or the combination of both) (*p* = 0.104), creation of ileostomy (*p* = 0.547), creation of ileoanal pouch (*p* = 0.364), and intraoperative findings such as a free perforation (*p* = 0.176) and serosal tear (*p* = 0.309) did not differ between the cohorts. There were more contaminated (III) and dirty (IV) wounds during the COVID-19 pandemic: twenty-three (23.7%) vs. twelve (11.2%) class III, and three (3.1%) vs. two (1.9%) class IV; *p* = 0.047. There was a trend towards more intraoperatively detected intra-abdominal abscesses during the COVID-19 pandemic: eleven (11.3%) patients vs. five (4.7%) patients pre-COVID-19 (*p* = 0.077). Furthermore, there was a trend towards more transmural bowel lesions during the COVID-19 pandemic: seven (7.2%) vs. two (1.9%) patients (*p* = 0.063). 

### 3.3. Hygienic Restrictions during COVID-19 Pandemic

The regulations during the COVID-19 pandemic were categorized into “mild, medium, and strict” according to the imposed bans. In 70 patients (56.9%), surgery was performed when mild regulations were active; in 32 (26%) patients, during medium; and in 21 (17.1%) during strict bans. Six (18.8%) patients developed SSIs during the time of strict hygienic regulations (between the 17th of December 2020 and the 1st of April 2021) compared to 15 (16.5%) without SSI; *p* = 0.553. The strictness of hygienic measures (mild, medium, strict) during the COVID-19 pandemic had no influence on the rate of SSI (*p* = 0.553). 

**Figure 1:**
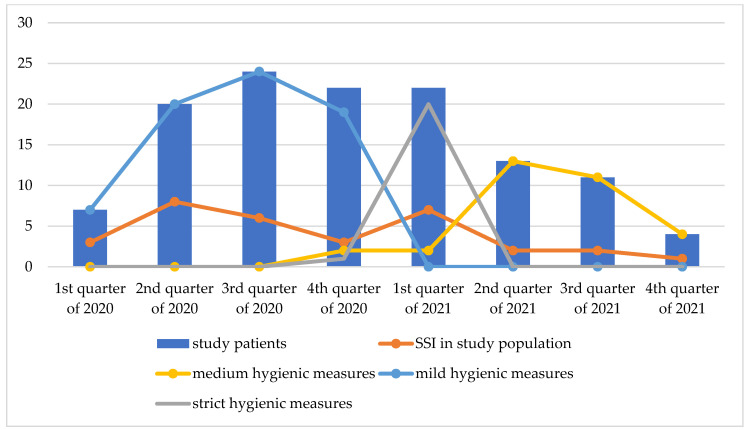
SSI and changes in preventive hygienic measures during the COVID-19 pandemic at Charité—Universitätsmedizin Berlin. Absolute numbers; SSI, surgical site infection; the y-axis represents the absolute number of patients; the x-axis shows the different quarters of 2020 and 2021.

Figure 1 depicts the rate of SSI in our study during the different quarters of 2020 and 2021 and the chronological sequence of preventive hygienic measures A downward trend in the SSI rate was observed between the third and fourth quarters of 2020. At that time, medium, followed by strict hygienic restrictions, had been active since the 17th of December 2020. However, in the wake of strict hygienic regulations in the first quarter of 2021, there was an upward trend in SSIs in our study population. The rate of SSI dropped again after the second quarter of 2021, with medium restrictions in place. 

### 3.4. Postoperative Complications during the COVID-19 Pandemic and Pre-COVID-19

There was no difference in severe postoperative complications according to Clavien–Dindo 3–5 (*p* = 0.181) and the rate of reoperation (*p* = 0.541) between pre-COVID-19 and COVID-19 (Table 4). The median duration of surgery was longer during the COVID-19 pandemic: 3.58 (1.26–8.46) hours vs. 3.23 (0.58–9.46) hours; *p* = 0.003. The median length of hospital stay was significantly shorter during the COVID-19 pandemic: 8.0 (3–64) days during COVID-19 vs. 11.0 (4–43) days pre-COVID-19; *p* ≤ 0.001. 

The rate of postoperative, intra-abdominal abscesses was higher during the COVID-19 pandemic than pre-COVID-19: seven patients (7.2%) vs. one patient (0.9%); *p* = 0.021. Looking only at the COVID-19 cohort, postoperative intra-abdominal abscesses occurred more frequently in patients with SSI than without SSI: eight (25%) vs. two (2.2%); *p* < 0.001. Patients with postoperative abscesses were more likely to be underweight (BMI < 18.5 kg/m^2^): four (40%) vs. eleven (10%); *p* = 0.015. Patients with wound class IV (dirty) had, more often, postoperative intra-abdominal abscesses, when only considering the COVID-19 cohort: three (30%) vs. eight (7.1%) patients; *p* = 0.05. There is information about preoperative albumin levels in six out of ten patients with postoperative abscesses during the COVID-19 pandemic. All six patients who developed postoperative abscesses had preoperative hypoalbuminemia. 

### 3.5. Patient Characteristics of Study Population during the COVID-19 Pandemic

Overall, 214 patients were eligible to participate in the study. The COVID-19 era was defined as the period between the 1st of March 2020 and the 15th of December 2021. One hundred and twenty-three patients answered the questionnaire and therefore completed the follow-up of 30 days postoperatively. The response rate to the questionnaire was 57.5%. The baseline characteristics, urgency of operation (*p* = 0.139), and postoperative complications according to Clavien–Dindo 3–5 (*p* = 0.284) did not differ between patients who completed the follow-up and those who did not answer or disagreed with participation (see Table 5). 

### 3.6. First and Second Waves of the COVID-19 Pandemic in Germany

In Germany, the first wave of the COVID-19 pandemic was described between March and May 2020. The second wave started at the end of September 2020 and lasted until the end of February 2021. Hygienic regulations at Charité first became stricter in the middle of December 2020. Medium and strict hygienic measures, as defined in our retrospective trial, were present during the second wave in Germany. Table 6 shows study participants during the first and second waves of the COVID-19 pandemic, a total of 61 patients. On univariate analysis, there were more SSIs during the first wave compared to the second wave: nine (50%) versus nine (20.9%); *p* = 0.023. Wound status did not differ; *p* = 0.098. 

## 4. Discussion

The main finding of this monocentric, retrospective study was that despite a lower SSI rate (25.8%) for elective bowel resections for IBD during the COVID-19 pandemic compared with the pre-COVID-19 era (31.8%), no significant difference in SSI rate was demonstrated. The strictness of hygienic measures (mild, medium, strict) during the COVID-19 pandemic had no influence on the rate of SSI in our trial. In fact, the rate of postoperatively detected intraabdominal abscesses was higher during the COVID-19 pandemic than the pre-COVID-19 in our trial.

In our study, patients during the COVID-19 era had higher ASA scores than pre-COVID-19 and were, therefore, more often critically ill. This could be due to a greater disease activity in IBD because of the postponement of surgery during the operation ban during the COVID-19 pandemic. An ASA score of 3–4 was associated with more SSIs, as shown in previous trials [7,25]. A higher ASA score was also related to reoperation and severe postoperative complications according to Clavien–Dindo 3–5 in our trial. Therefore, higher ASA scores and related higher risks for SSI during the COVID-19 pandemic impeded the effect of hygienic regulations on the rate of SSI. Furthermore, the duration of surgery was longer during the COVID-19 pandemic than pre-COVID-19, which is a known risk factor for SSI [7]. 

Deep incisional SSIs and organ/space SSIs occurred more frequently during COVID-19 (4.1%) compared to pre-COVID-19 (1.9%), respectively, without statistical significance. A multicenter trial in the United States of America displayed a trend towards more deep incisional SSI in colorectal surgery during COVID-19 [26]. A monocentric observational cohort study comparing 41 patients with colorectal surgery during COVID-19 (March to April 2020) to 164 patients pre-COVID-19 showed fewer organ–space SSIs during COVID-19 [27].

The rate of postoperatively detected intra-abdominal abscesses was higher during the COVID-19 pandemic than before (RECIPE trial). One explanation is that there were more patients with contaminated (III) and dirty (IV) wounds, thus intra-abdominal abscesses or perforation, during COVID-19 than pre-COVID-19. Wound class IV was a risk factor for postoperative intra-abdominal abscess in our cohort during the COVID-19 pandemic. It is well known that contaminated or dirty wounds are predictors of increased SSI rates [7]. There was a slight trend towards more contaminated (III) but fewer dirty wounds (IV) in colorectal surgery during COVID-19 in a retrospective multicenter analysis [26].

Patients with IBD, especially Crohn’s disease, are more likely to develop SSI than patients with colorectal cancer or diverticulitis [8]. Furthermore, enteric fistula or abscesses often develop during the disease course. The operation ban during the COVID-19 pandemic may have led to a delay in surgical therapy and therefore deterioration in intra-abdominal findings. Furthermore, the nutritional status of patients with IBD can be impaired and worsen throughout disease progression [8,28]. In our study, hypoalbuminemia was present in 30 (42.98%) of 70 patients with available preoperative data. All six patients who developed postoperative abscesses had hypoalbuminemia. 

A risk reduction in SSI during the COVID-19 pandemic was shown in two single-center studies in colorectal oncological surgery [27,29] and in one trial based on national surveillance data in colorectal surgery [30]. The number of patients was limited in the single-center studies, and elective surgery was conducted only in 72%. A difference to our trial is the definition of the period of the pandemic, which in our case was longer, and the associated measures also took place later. During the first wave of the pandemic in Germany—March to May 2020—the only prohibition at Charité was having more than one visitor per patient per day. Hygienic regulations at Charité became stricter first in the middle of December 2020 during the second wave of COVID-19 in Germany [31]. In our monocentric, retrospective cohort study, there were more SSIs during the first wave of the pandemic compared to the second wave in Germany. So, there might be an effect of the stricter hygienic regulations at Charité on the rate of SSI. There is one recently published study supporting our findings of no reduction in SSI rate during COVID-19. In this study, 112 patients with colorectal surgery (out of 29,904 general surgeries) were compared in a matched analysis. However, no IBD was reported [26].

Procedure- and patient-related risk factors for SSI might have impeded the effect of hygienic regulations during COVID-19 on the rate of SSI. Furthermore, the primary intention of hygienic regulations in hospitals during the COVID-19 pandemic was not to reduce SSIs but to prevent the spreading of SARS-CoV2. Compliance with perioperative antiseptic guidelines by WHO is crucial in preventing SSI [19,20]. These include correct perioperative antibiotic prophylaxis. Incorrect, non-procedure-specific selection and timing of perioperative antibiotics led to a 2.9 greater odds for SSI in a retrospective cohort study in the United States of America between January 2020 and September 2021 [32].

Several potential limitations of the study need to be considered. First, this was a monocentric, retrospective study. Data about the patients during the COVID-19 pandemic were extracted retrospectively, and the response rate to the questionnaire concerning the rate of SSI 30 days postoperatively was 57.5%. The onset of postoperative complications had no influence on the response rate to the questionnaire during follow-up, but the analyzed cohort of 97 patients with IBD during the COVID-19 pandemic is only a small number. A risk reduction in the SSI rate would probably be evident in a larger study population. Second, there was no clinical examination of the patients 30 days postoperatively, leaving the chance of missing some SSIs. Follow-up comprised a written questionnaire completed by all the patients retrospectively 30 days postoperatively.

Third, there is a difference in intraoperative, subcutaneous wound irrigation due to the study design of the RECIPE trial pre-COVID-19. These patients participated in the randomized controlled prospective study with two parallel treatment groups comparing intraoperative, subcutaneous wound irrigation with 0.04% polyhexanide solution to 0.9% saline. The risk reduction in SSI in the RECIPE trial led to the implementation of 0.04% polyhexanide solution as a standard for subcutaneous wound irrigation in our department. Analysis of patients flushed subcutaneously with a 0.04% polyhexanide solution before the COVID-19 pandemic and during the COVID-19 pandemic showed a similar rate of SSI (25.4% vs. 25.8%). 

## 5. Conclusions

The hygienic regulations during the COVID-19 pandemic at Charité (operation ban, visiting ban, wearing of FFP2 mask, social distance, disinfection) did not significantly reduce the rate of SSI in patients with bowel resection due to inflammatory bowel disease. A risk reduction in the SSI rate could perhaps be evident in a larger cohort.

Patient-related risk factors for SSI play an important role. It is crucial to optimize preoperative status including normal weight, good nutritional status, correction of anemia and cessation of smoking in elective surgery. Hygienic regulations in hospitals during the COVID-19 pandemic did not have an influence on these patient-related risk factors. A ban on surgery, where only emergency surgery was allowed, was likely to delay surgery and exacerbate the disease, which probably contributed to more SSIs and postoperative complications. Compliance with perioperative antiseptic guidelines by WHO is crucial in preventing SSIs.

## Figures and Tables

**Table 1:** Definition of Surgical Site Infection (SSI) according to the Centers for Disease Control and Prevention (CDC) [21].

Superficial incisional SSI	Infection occurs within 30 days after the operation AND Involves only skin and subcutaneous tissue of the incision AND the patient has at least one of the following: 1. Purulent drainage from the superficial incision. 2. Organisms isolated from an aseptically obtained culture of fluid or tissue from the superficial incision. 3. At least one of the following symptoms: localized pain or tenderness; localized swelling; erythema; or heat and the superficial incision is deliberately opened by the surgeon unless the incision is culture-negative. 4. Diagnosis of superficial incisional SSI by the surgeon.
Deep incisional SSI	Infection occurs within 30 days after the operation, and the infection appears to be related to the operation AND Involves deep soft tissue of the incision (e.g., fascial and muscle layers), AND the patient has at least one of the following: 1. Purulent drainage from the deep incision but not from the organ/space component of the surgical site. 2. A deep incision spontaneously dehisces or is deliberately opened by a surgeon when the patient has at least one of the following symptoms: fever (>38 °C); localized pain or tenderness; unless the site is culture-negative. 3. An abscess or other evidence of infection involving the deep incision is found on direct examination, during reoperation, or by histopathologic or radiologic examination. 4. Diagnosis of a deep incisional SSI by a surgeon.
Organ/Space SSI	Infection occurs within 30 days after the operation, and the infection appears to be related to the operation, AND Involves any parts of the body deeper than fascial/muscle layers that were opened or manipulated during an operation, AND the patient has at least *one* of the following: 1. Purulent drainage from a drain that was placed into the organ/space. 2. Organisms isolated from an aseptically obtained culture of fluid or tissue in the organ/space. 3. An abscess or other evidence of infection involving the organ/space is found on direct examination, during reoperation, or by histopathologic or radiologic examination. 4. Diagnosis of an organ/space SSI by a surgeon.

**Table 2:** Surgical Site Infections of Cohort in COVID-19 Era and Pre-COVID-19.

	COVID-19 Era (n = 97)	Pre-COVID-19 Era (n = 107)	Total (n = 204)	*p*-Value
SSI	25 (25.8%)	34 (31.8%)	59 (28.9%)	0.345 §
CDC Type				0.216 §
Superficial incisional SSI	17 (17.5%)	30 (28.0%)	47 (23.0%)	
Deep incisional SSI	4 (4.1%)	2 (1.9%)	6 (2.9%)	
Organ/Space SSI	4 (4.1%)	2 (1.9%)	6 (2.9%)	
Wound irrigation				<0.001 *§
Saline	0 (0%)	48 (44.9%)	48 (23.5%)	
Polihexanide	97 (100%)	59 (55.1%)	156 (76.5%)	
Wound status				0.047 *§
Clean (I)	0 (0%)	0 (0%)	0 (0%)	
Clean-contaminated (II)	71 (73.2%)	93 (86.9%)	164 (80.4%)	
Contaminated (III)	23 (23.7%)	12 (11.2%)	35 (17.2%)	
Dirty (IV)	3 (3.1%)	2 (1.9%)	5 (2.4%)	

Data were n (%); SSI, surgical site infection; §, chi-square test; CDC, Centers for Disease Control and Prevention; * *p* ≤ 0.05.

**Table 3:** Baseline Characteristics of Cohort in COVID-19 Era and Pre-COVID-19.

	COVID-19 Era (n = 97)	Pre-COVID-19 Era (n = 107)	Total (n = 204)	*p*-Value
Sex				0.276 §
Female	50 (51.5%)	47 (43.9%)	97 (47.5%)	
Male	47 (48.5%)	60 (56.1%)	107 (52.5%)	
Age (years)	40.0 (19–91)	41.0 (20–80)	40.0 (19–91)	0.790 †
BMI (kg/m^2^) (n = 203)	23.5 (15.2–38.2)	22.3 (15.2–35.6)	22.8 (15.2–38.2)	0.179 †
Weight				0.634 §
Underweight	10 (10.4%)	10 (9.3%)	20 (9.9%)	
Normal weight	52 (54.2%)	65 (60.7%)	117 (57.6%)	
Overweight	34 (35.4%)	32 (30.0%)	66 (32.5%)	
IBD				0.864 §
Crohn’s disease	52 (53.6%)	60 (56.1%)	112 (54.9%)	
Ulcerative colitis	43 (44.3%)	44 (41.1%)	87 (42.6%)	
Colitis indeterminata	2 (2.1%)	3 (2.8%)	5 (2.5%)	
ASA score				0.021 *§
1–2	72 (74.2%)	93 (86.9%)	165 (80.9%)	
3–4	25 (25.8%)	14 (13.1%)	39 (19.1%)	
Comorbidities				
Coronary artery disease	4 (4.1%)	5 (4.7%)	9 (4.4%)	0.849 §
Liver cirrhosis	1 (1.0%)	1 (0.9%)	2 (1.0%)	0.944 §
Chronic pulmonary disease	3 (3.1%)	0 (0%)	3 (1.5%)	0.067 §
Diabetes mellitus	2 (2.1%)	9 (8.4%)	11 (5.4%)	0.045 *§
Malignant disease	16 (16.5%)	7 (6.5%)	23 (11.3%)	0.025 *§
Renal insufficiency	4 (4.1%)	6 (5.6%)	10 (4.9%)	0.624 §
Nicotine abuse (n = 202)	16 (16.8%)	25 (23.4%)	41 (20.3%)	0.250 §
Anemia (n = 202)	30 (31.6%)	52 (48.6%)	82 (40.6%)	0.014 *§
Current immunosuppressive medication	58 (59.8%)	67 (62.6%)	125 (61.3%)	0.679 §
Prior abdominal operation	64 (66.0%)	71 (66.4%)	135 (66.2%)	0.955 §

Data were n (%) or median, minimum—maximum; §, chi-square test; * *p* ≤ 0.05; †, Mann–Whitney U test; BMI, body mass index; underweight BMI, <18.5 kg/m^2^; normal weight BMI, 18.5–25 kg/m^2^; overweight BMI, >25 kg/m^2^; IBD, inflammatory bowel disease; ASA, American Society of Anesthesiology; renal insufficiency, creatinine > 0.9 mg/dL in females and >1.2 mg/dL in males; anemia, hemoglobin < 12.0 g/dl in females and <13.5 g/dL in males; current treatment, within six weeks before operation; immunosuppressive medication, including glucocorticoids, methotrexate, azathioprine, and biologicals.

**Table 4:** Surgical Characteristics of Cohort in COVID-19 Era and Pre-COVID-19.

	COVID-19 Era (n = 97)	Pre-COVID-19 Era (n = 107)	Total (n = 204)	*p*-Value
Resected organ				0.104 §
Small bowel and colon	37 (38.1%)	38 (35.5%)	75 (36.8%)	
Small bowel	11 (11.4%)	24 (22.4%)	35 (17.1%)	
Colon	49 (50.5%)	45 (42.1%)	94 (46.1%)	
Creation of ileostomy	62 (63.9%)	64 (59.8%)	126 (61.8%)	0.547 §
Creation of ileoanal pouch	16 (16.5%)	23 (21.5%)	39 (19.1%)	0.364 §
Surgical approach				0.479 §
Minimally invasive	54 (55.7%)	55 (51.4%)	109 (53.4%)	
Open	28 (28.8%)	39 (36.4%)	67 (32.8%)	
Conversion	15 (15.5%)	13 (12.2%)	28 (13.8%)	
Duration of surgery (hours)	3.58 (1.26–8.46)	3.23 (0.58–9.46)	3.41 (0.58–9.46)	0.003 *†
Intraoperative findings				
Free perforation	0 (0%)	2 (1.9%)	2 (1.0%)	0.176 §
Covered perforation	2 (2.1%)	0 (0%)	2 (1.0%)	0.136 §
Intraoperative findings				
Intra-abdominal abscess	11 (11.3%)	5 (4.7%)	16 (7.8%)	0.077 §
Intraoperative transmural bowel lesion	7 (7.2%)	2 (1.9%)	9 (4.4%)	0.063 §
Intraoperative serosal tear	4 (4.1%)	8 (7.5%)	12 (5.9%)	0.309 §
Length of hospital stay (days)	8.0 (3–64)	11.0 (4–43)	9.0 (3–64)	<0.001 *†
Postoperative complications				
Bleeding	3 (3.1%)	5 (4.7%)	8 (3.9%)	0.561 §
Haematoma	2 (2.1%)	5 (4.7%)	7 (3.4%)	0.306 §
Ileus	1 (1.0%)	3 (2.8%)	4 (2.0%)	0.362 §
Dehiscence of fascia	3 (3.1%)	2 (1.9%)	5 (2.5%)	0.572 §
Postoperative complications				
Abscess	7 (7.2%)	1 (0.9%)	8 (3.9%)	0.021 *§
Insufficiency of anastomosis	4 (4.1%)	8 (7.5%)	12 (5.9%)	0.309 §
Clavien–Dindo 3–5	24 (24.7%)	21 (19.6%)	45 (22.1%)	0.181 §
Reoperation	15 (15.5%)	20 (18.7%)	35 (17.2%)	0.541 §
Reoperation				0.364 §
Because of SSI	4 (4.1%)	2 (1.9%)	6 (2.9%)	
Other reason	11 (11.3%)	18 (16.8%)	29 (14.2%)	

Data were n (%) or median, minimum—maximum; §, chi-square test; * *p* ≤ 0.05; †, Mann Whitney U test.

**Table 5:** Baseline Characteristics of Patients With and Without Completed 30-Day Follow-Up during COVID-19 Pandemic.

	Follow-Up (n = 123)	No Follow-Up (n = 90)	Total (n = 213)	*p*-Value
Sex				0.875 §
Female	67 (54.5%)	50 (55.6%)	117 (54.9%)	
Male	56 (45.5%)	40 (44.4%)	96 (45.1%)	
Age (years)	41.0 (19–91)	35.5 (18–79)	39.0 (18–91)	0.060 †
BMI (kg/m^2^) (n = 208)	23.1 (15.3–38.2)	23.1 (14.9–43.3)	23.1 (14.9–43.3)	0.967 †
ASA score				0.914 §
1–2	88 (71.5%)	65 (72.2%)	153 (71.8%)	
3–4	35 (28.5%)	25 (27.8%)	60 (28.2%)	
IBD				0.237 §
Crohn’s disease	71 (57.7%)	60 (66.7%)	131 (61.5%)	
Ulcerative colitis	50 (40.7%)	30 (33.3%)	80 (37.6%)	
Colitis indeterminata	2 (1.6%)	0 (0%)	2 (0.9%)	
Urgency				0.139 §
Elective	112 (91.1%)	76 (84.4%)	188 (88.3%)	
Emergency	11 (8.9%)	14 (15.6%)	25 (11.7%)	
Postoperative complications Clavien–Dindo 3–5	33 (26.8%)	27 (30.0%)	60 (28.2%)	0.284 §

Data were n (%) or median, minimum—maximum; §, chi-square test; †, Mann–Whitney U test; BMI, body mass index; ASA, American Society of Anesthesiology; IBD, inflammatory bowel disease.

**Table 6:** Surgical Site Infection in First and Second Waves of COVID-19 Pandemic in Germany.

	First Wave (n = 18)	Second Wave (n = 43)	Total (n = 61)	*p*-Value
SSI	9 (50.0%)	9 (20.9%)	18 (29.5%)	0.023 *§
CDC Type				0.129 §
Superficial incisional	6 (33.3%)	5 (11.6%)	11 (18.0%)	
Deep incisional	1 (5.6%)	2 (4.7%)	3 (4.9%)	
Organ/Space SSI	2 (11.1%)	2 (4.7%)	4 (6.6%)	
Wound status				0.098 §
Clean-contaminated (II)	10 (55.6%)	32 (74.4%)	42 (68.9%)	
Contaminated (III)	4 (22.2%)	9 (20.9%)	13 (21.3%)	
Dirty (IV)	4 (22.2%)	2 (4.7%)	6 (9.8%)	

Data were n (%); SSI, surgical site infection; §, chi-square test; CDC, Center for Disease Control and Prevention; * *p* ≤ 0.05.

## Data Availability

Dataset available on request from the authors.

## Abbreviations

COVID-19	Coronavirus Disease 2019
COI	conflict of interest
SSI	surgical site infection
IBD	inflammatory bowel disease
RECIPE	Reduction of Postoperative Wound Infections by Antiseptica?
OR	odds ratio
CI	confidence interval
WHO	World Health Organization
SARS-CoV-2	Severe acute respiratory syndrome coronavirus 2
USD	U.S. Dollar
CDC	Centers for Disease Control and Prevention
BMI	body mass index
ASA	American Society of Anesthesiology
